# EpiGraph: an open-source platform to quantify epithelial organization

**DOI:** 10.1093/bioinformatics/btz683

**Published:** 2019-09-06

**Authors:** Pablo Vicente-Munuera, Pedro Gómez-Gálvez, Robert J Tetley, Cristina Forja, Antonio Tagua, Marta Letrán, Melda Tozluoglu, Yanlan Mao, Luis M Escudero

**Affiliations:** 1 Instituto de Biomedicina de Sevilla (IBiS), Hospital Universitario Virgen del Rocío/CSIC/Universidad de Sevilla and Departamento de Biología Celular, Universidad de Sevilla, Seville 41013, Spain; 2 Biomedical Network Research Centre on Neurodegenerative Diseases (CIBERNED), Madrid 28031, Spain; 3 MRC Laboratory for Molecular Cell Biology, University College London, London WC1E 6BT, UK; 4 College of Information and Control, Nanjing University of Information Science and Technology, Nanjing, Jiangsu 210044, China

## Abstract

**Summary:**

Here we present EpiGraph, an image analysis tool that quantifies epithelial organization. Our method combines computational geometry and graph theory to measure the degree of order of any packed tissue. EpiGraph goes beyond the traditional polygon distribution analysis, capturing other organizational traits that improve the characterization of epithelia. EpiGraph can objectively compare the rearrangements of epithelial cells during development and homeostasis to quantify how the global ensemble is affected. Importantly, it has been implemented in the open-access platform Fiji. This makes EpiGraph very user friendly, with no programming skills required.

**Availability and implementation:**

EpiGraph is available at https://imagej.net/EpiGraph and the code is accessible (https://github.com/ComplexOrganizationOfLivingMatter/Epigraph) under GPLv3 license.

**Supplementary information:**

Supplementary data are available at *Bioinformatics* online.

## 1 Introduction

How tissues modulate and maintain their organization during development and homeostasis is an important question that remains unsolved. This is mainly due to the lack of simple and general methods that can capture and quantify the arrangement of cells. It has been known for almost a hundred years that epithelial tissues exhibit a degree of order. The analysis of epithelial organization has been mainly based on the number of neighbors of the epithelial cells, considering the apical surface of these cells as convex polygons with the same number of sides as neighbors. We have described that the polygon distribution of natural tessellations (Supplementary Box) is restricted to a series of frequencies of polygons that match the Voronoi diagrams (Supplementary Box) that conform to the Centroidal Voronoi tessellation (CVT). This is what we call a ‘CVT path’ (Supplementary Box), which was used as a scale to compare the organization of different packed tissues. However, polygon distribution is not sufficient to completely characterize tissue organization. Tissues with clearly different appearances can present very similar polygon distributions (Sanchez-Gutierrez *et al.*, 2016).

**Fig. 1.**
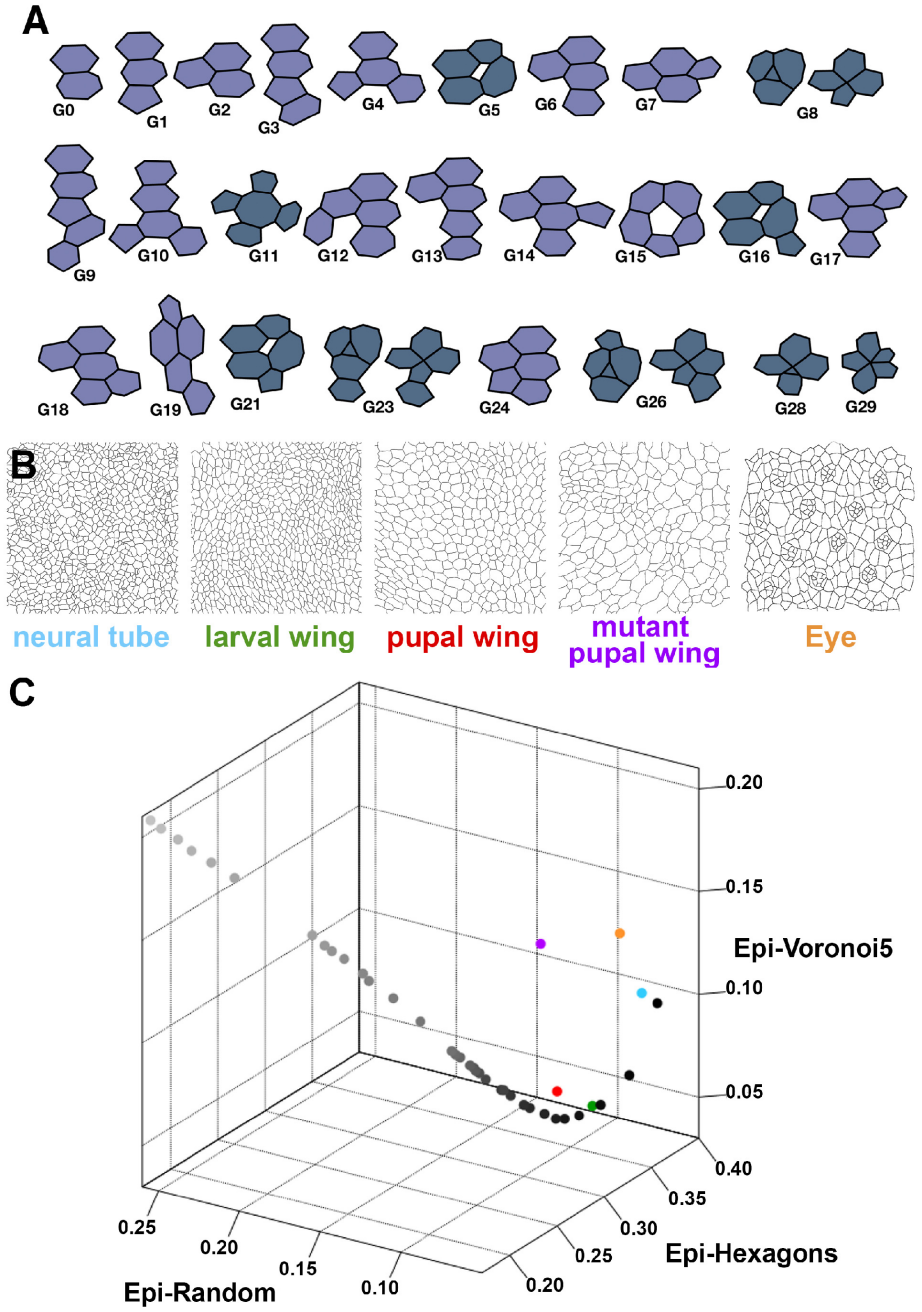
Graphlets, cellular motifs and characterization of epithelial organization. (**A**) A representation of the cellular motifs that correspond to graphlets of up to five nodes. There are 29 motifs corresponding to 26 different graphlets. Note that one graphlet can represent two cellular motifs (G8, G23 and G26). Epigraph allows the use of different sets of motifs. Mauve motifs form the 17-motif set that has been used in C. Prussian Blue indicates the remaining motifs. (**B**) Representative images from the sets of natural tessellations. (**C**) EpiGraph plots showing the distribution of the CVTn path and the average values obtained from the natural tessellation images. The CVTn is represented from iteration 1 until iteration 700 in grayscale, beginning in black and reducing its darkness with the increase of the iterations (from 1 to 20, from 30 to 100 in steps of 10 and from 100 to 700 in steps of 100). The axis of the graph corresponds to the values for the 17-motif set of Epi-Hexagons, Epi-Random and Epi-Voronoi5. The natural tessellations are larval wing (green), pupal wing (red), neural tube (light blue), mutant pupal wing (violet) and eye (orange)

Graph Theory has been used to capture and quantify the topology of tissues from histopathological images by using the cell nuclei as the nodes of a network (Gurcan *et al.*, 2009; MacAulay *et al.*, 2017). Furthermore, cell-graph approaches can even be designed to consider the extracellular matrix between the cells in an image (Bilgin *et al.*, 2009). In contrast, the study of epithelial organization in development has instead been primarily based on the detection of cell outlines (Classen *et al.*, 2005; Gibson *et al.*, 2006), allowing the generation of a network of true cell-cell contacts (Escudero *et al.*, 2011; Sanchez-Gutierrez *et al.*, 2013), rather than inferred contacts using the cell nuclei approach. A network can be split up into different subgraphs named graphlets (Supplementary Box). The graphlet composition of a network has been used to quantify differences between complex systems (Hayes *et al.*, 2013; Ho *et al.*, 2010; Kuchaiev *et al.*, 2011; Pržulj *et al.*, 2004). This method implied calculating the Graphlet degree Distribution agreement Distance (GDD) (Supplementary Box) between two networks (Pržulj, 2007). The ‘GDD value’ weighs the differences among the two distributions of graphlets; the higher the value, the more different the arrangements (Supplementary Fig. S1). These measurements are based on the comparison of the quantity of each subgraph in different networks, providing an index of distance between them. This feature has the advantage of integrating the differences between diverse networks into a single value, simplifying the analyses and allowing multiple comparisons (Fig. 1). Here we present an open source platform, EpiGraph, a new image analysis method that uses segmented images from real epithelia or simulations, to easily quantify and compare the organization of packed tissues.

## 2 Materials and methods

– Example epithelial tissues

The details of the obtaining and processing the epithelial images are described in Escudero *et al.* (2011) and Sanchez-Gutierrez *et al.* (2016).

– Centroidal Voronoi Tessellation noise (CVTn) diagrams

We have developed a Voronoi scale named the CVT noise (CVTn) path (Supplementary Fig. S2, Supplementary Box). This approach is a variation of the CVT path (Sanchez-Gutierrez *et al.*, 2016). Beginning with seeds randomly placed, we created a Voronoi diagram and then applied a variation to the Lloyd algorithm (Lloyd, 1957) (Supplementary Box). In even iterations, we selected a region of 5 pixels in radius from the centroid position of the cells, in which seeds could be placed randomly. In odd iterations, the system was stabilized, applying the original Lloyd algorithm (Supplementary Fig. S2).

– Graphlet and motif selection

The different images from the previous two sections were used to create a graph of cell-to-cell contacts (Escudero *et al.*, 2011) that served as the source for the graphlet analysis (Pržulj *et al.*, 2004; Pržulj, 2007). We used the computer program ORCA (Orbit Counting Algorithm) for graphlet identification and calculation (Hočevar and Demšar, 2014) to extract the different conformations of nodes assembling the graphlets, called orbits (Pržulj, 2007). We computed the Graphlet degree Distribution of the 73 given orbits from the 29 graphlets and then removed the non-applicable ones. The reason to remove these graphlets was that they were either redundant or not possible in a planar tissue.

## 3 Results

### 3.1 EpiGraph quantitatively compares the organization of multiple sets of images using graphlets

Using the principle that an epithelial image can be converted into a cell-to-cell contact network, we identified the ‘cellular motifs’ that corresponded to graphlets of up to five nodes (Fig. 1A). In this way, we adapted the graphlet analysis to the nature of our samples. We designed EpiGraph, a Fiji plugin (Schindelin *et al.*, 2012) that calculates the GDD of any epithelial tissue with another tessellation that serves as a reference (Supplementary Fig. S1). We used three different references: (i) a tessellation formed by regular hexagons, representing the most ordered way to pave the space (Epi-Hexagons, Supplementary Box). (ii) The network motifs emerging from a random Voronoi tessellation (Epi-Random, Supplementary Box). (iii) A Voronoi Diagram 5 from the CVT path (Epi-Voronoi5, Supplementary Box) that presents a polygon distribution similar to the one from multiple examples in nature (Gibson *et al.*, 2006; Sanchez-Gutierrez *et al.*, 2016).

We tested the method with epithelial images that have been previously compared with the CVT path in terms of polygon distribution: chicken neural tube (neural tube), Drosophila larval wing disc (larval wing), Drosophila prepupal wing disc (pupal wing), reduction of myosin II in the Drosophila prepupal wing disc epithelium (mutant pupal wing) and Drosophila larval eye disc (Eye) (Fig. 1B) (Sanchez-Gutierrez *et al.*, 2016). To have a scale and facilitate fast comparisons, we used the concept of the CVTn path (see Material and methods). EpiGraph will integrate the values of Epi-Hexagons, Epi-Random and Epi-Voronoi5 in one plot to capture, in a single point, the organizational cues of a tessellation. These three coordinates compare the differences between the natural image and the CVTn scale (Fig. 1C, Supplementary Fig. S3).

In the case of neural tube, larval wing and pupal wing, the Epi-Hexagons, Epi-Random and Epi-Voronoi5 values were similar to the CVTn. However, the Eye and mutant pupal wing images presented a clear deviation. These results suggested that EpiGraph is able to distinguish between different tessellations with a similar polygon distribution (such as Voronoi Diagram 1, neural tube and Eye images). In this regard, we have developed a statistical output using an outlier detection approach whose quantitative results represent how similar the organization of a tissue is when compared with the CVTn scale. The test confirmed that neural tube, larval wing and pupal wing were close to the CVTn and similar to the Voronoi diagrams 1, 3 and 7, respectively. In contrast, the Eye and mutant pupal wing samples were labeled as different. In this way, EpiGraph provides a quantitative description of tissue organization.

In summary, we have generated a very accessible, open source method to produce a quantitative description of tissue organization in diverse epithelia. More examples of possible applications can be consulted in Vicente-Munuera *et al.* (2018). More details are provided at https://imagej.net/EpiGraph. We anticipate that our tool will improve the study morphogenesis by permitting the comparative analysis of epithelial organization in genetically mutated or diseased tissues in time lapse analyses.

## Funding

L.M.E. and P.G.-G. were supported by the Ramón y Cajal program (PI13/01347); L.M.E, P.V.-M. and P.G.-G. work was funded by the Ministry of Economy, Industry and Competitiveness grant BFU2016-74975-P co-funded by FEDER funds. P.V.-M. was supported by a contract co-funded by the Asociación Fundación Española contra el Cáncer and the Seville University. A.T. and C.F. were supported by a contract from Sistema Nacional de Garantía Juvenil and Programa Operativo de Empleo Juvenil 2014-2020. R.J.T. was funded by a Medical Research Council Skills Development Fellowship (MR/N014529/1). Y.M. was funded by a Medical Research Council Fellowship (MR/L009056/1), a UCL Excellence Fellowship, a NSFC International Young Scientist Fellowship (31650110472) and a Lister Institute Research Prize Fellowship. This work was also supported by MRC funding to the MRC LMCB University Unit at UCL (award code MC_U12266B). M.T. was funded by a Sir Henry Wellcome Fellowship (Grant No: 103095).

*Conflict of Interest*: none declared.

## Supplementary Material

btz683_Supplementary_Data
