# Supplementary material for: EpiGraph: an open-source platform to quantify epithelial organization
Source: Bioinformatics. 2019 Sep 6;36(4):1314–6. doi: 10.1093/bioinformatics/btz683 (PMC7703762; doi:10.1093/bioinformatics/btz683)
Supplement: btz683_Supplementary_Data [file btz683_supplementary_data.zip › Vicente-Munuera Supplementary Information.docx]

**EpiGraph: an open-source platform to quantify epithelial organization.**

**Supplementary Information.**

**SUPPLEMENTARY FIGURES**

**Supplementary Figure 1. Pipeline for the Graphlet degree Distribution agreement Distance (GDD) calculation.** Scheme representing the protocol sequence to calculate the GDD between a segmented image (Eye) and three references (Hexagonal lattice, Voronoi 1 diagram and Voronoi 5 diagram). First, a network of cell-to-cell contacts is computed defining the centroids of valid cells as nodes and their connections with neighbouring cells as edges. The light blue edges in these panels represent the cellular connectivity network. The coloured nodes mark the valid cells that were included in the cellular motifs used for graphlet quantification. The blue nodes are the 4-distance valid cells (cells connected exclusively to valid cells within a distance of 4 edges) used to quantify the graphlets for the 17-motif set. Cells without nodes could not be used for graphlet calculation. Second, the graphlets are extracted from the network, allowing the calculation of each graphlet’s frequency. The same protocol is carried out to obtain the graphlet degree distribution in 20 samples of Voronoi 1 diagrams, Voronoi 5 diagrams and hexagonal lattices, which are used as our three references. Finally, a comparison is made between the Graphlet degree Distributions of the source image and each reference, to obtain the final three GDD distance values: Epi-Hexagons, Epi-Random and Epi-Voronoi5.

**Supplementary Figure 2. CVTn scale capturing tissue organization.** Representation showing how the CVTn iteratively evolves (**methods and Supplementary Box**) from a disordered and heterogeneous tessellation (V1) to a more homogeneous and uniform Voronoi (V700). Each diagram is presented in circles surrounded with greyscale tones. The series of dots between the circles represent the middle diagrams amid V4 and V700. CVTn, based on the original CVT path**,** has been shown to be a good descriptor for identifying a tissue in homeostatic state. The graphic displays the resulting relationship between the natural samples and their most akin Voronoi diagram (signed by a double arrow) in terms of organization computed by EpiGraph. In addition, it shows which biological samples were contained within the scale (IN) and which were not (OUT). The colour code of the actual samples is to the same as that in **Fig. 1B** and **C.**

**Supplementary Figure 3. Scatter plot of GDD values for the individual natural images.** Representation of the GDD values (Epi-Hexagons, Epi-Random and Epi-Voronoi5) of the individual biological tessellations: larval wing (green), pupal wing (red), neural tube (light blue), mutant larval wing (violet) and Eye (orange), displaying the CVTn scale (greyscale, from the iteration 1 (black) until the iteration 700 (lightest grey)). Note that the average of the individual cases for each type of natural tessellation have been displayed in **Fig. 1C.**

**Supplementary Movie 1. A general example of the usage of EpiGraph.** The movie shows all the functionalities of Epigraph and how to use them, in general terms.

**Supplementary Box**

**Tessellation**: bounded Euclidean space filled with geometrical pieces occupying all the space without allowing empty regions, building a mosaic where the tiles do not overlap between them.

**Voronoi diagram:** tessellation composed of polygons with convex shape. Each one of these polygons represents a Voronoi cell that emerges from a seed. A Voronoi cell is defined as a cell where all the coordinates into this region are closer to its generator seed than to any other seed (generator of another Voronoi cell).

**Lloyd algorithm**: an iterative method to construct a series of Voronoi tessellations that are progressively converging to a homogeneous hexagonal lattice. The process is structured in two steps. First, the centroids of the Voronoi cells from a given tessellation are calculated. Subsequently, a Voronoi diagram is generated by placing the seeds in the previously calculated centroids positions.

**CVT path:** scale composed by consecutive Lloyd iterations starting from a random Voronoi diagram. The Voronoi diagram built after the first Lloyd iteration will be named as Voronoi diagram 2, after the second iteration Voronoi diagram 3 and so on. Following this process, the initial irregular tessellation turns into a very homogeneous polygonal mosaic reaching a high percentage of hexagonal cells. The CVT path has been verified as a predictor of homeostatic biological tessellations by comparison of polygon distributions, where pathologic or mutant tissues could be captured as outliers.

**CVTn path:** Variation of CVT path called CVT “noise”. The CVTn path is generated using a modified method for positioning the seeds in successive iterations of the Lloyd algorithm. Specifically, a circular zone of 5 pixels of radius from each centroid of polygon was selected, where the seed could be chosen randomly. This alteration was only included in the even steps, meanwhile in odd iterations the standard Lloyd algorithm was applied.

**Graphlets**: subgraph with low number of nodes, extracted from a larger and more complex network. Any network can be described by its graphlet make-up.

**Graphlet degree Distribution agreement Distance (GDD):** measurement describing how similar two networks are by comparing the distribution of their graphlets. The possible values range between 0 (the images are very similar) and 1 (the images are totally different).

**Epi-hexagons:** Particular GDD measurement that quantifies the difference between a given image and a regular tessellation of hexagons.

**Epi-Random:** Average GDD between a given image and 20 Voronoi diagrams where its seeds have been placed in a random way (Voronoi diagrams 1).

**Epi-Voronoi5:** Average GDD between a given image and 20 Voronoi diagrams 5 (after 4 Lloyd iterations from Voronoi diagrams random). The polygon distribution of a Voronoi diagram 5 corresponds to the “conserved polygon distribution” observed in multiple examples of proliferating epithelia in nature.
