# Supplementary figures and images for: EpiGraph: an open-source platform to quantify epithelial organization
Source: Bioinformatics. 2019 Sep 6;36(4):1314–6. doi: 10.1093/bioinformatics/btz683 (PMC7703762; doi:10.1093/bioinformatics/btz683)

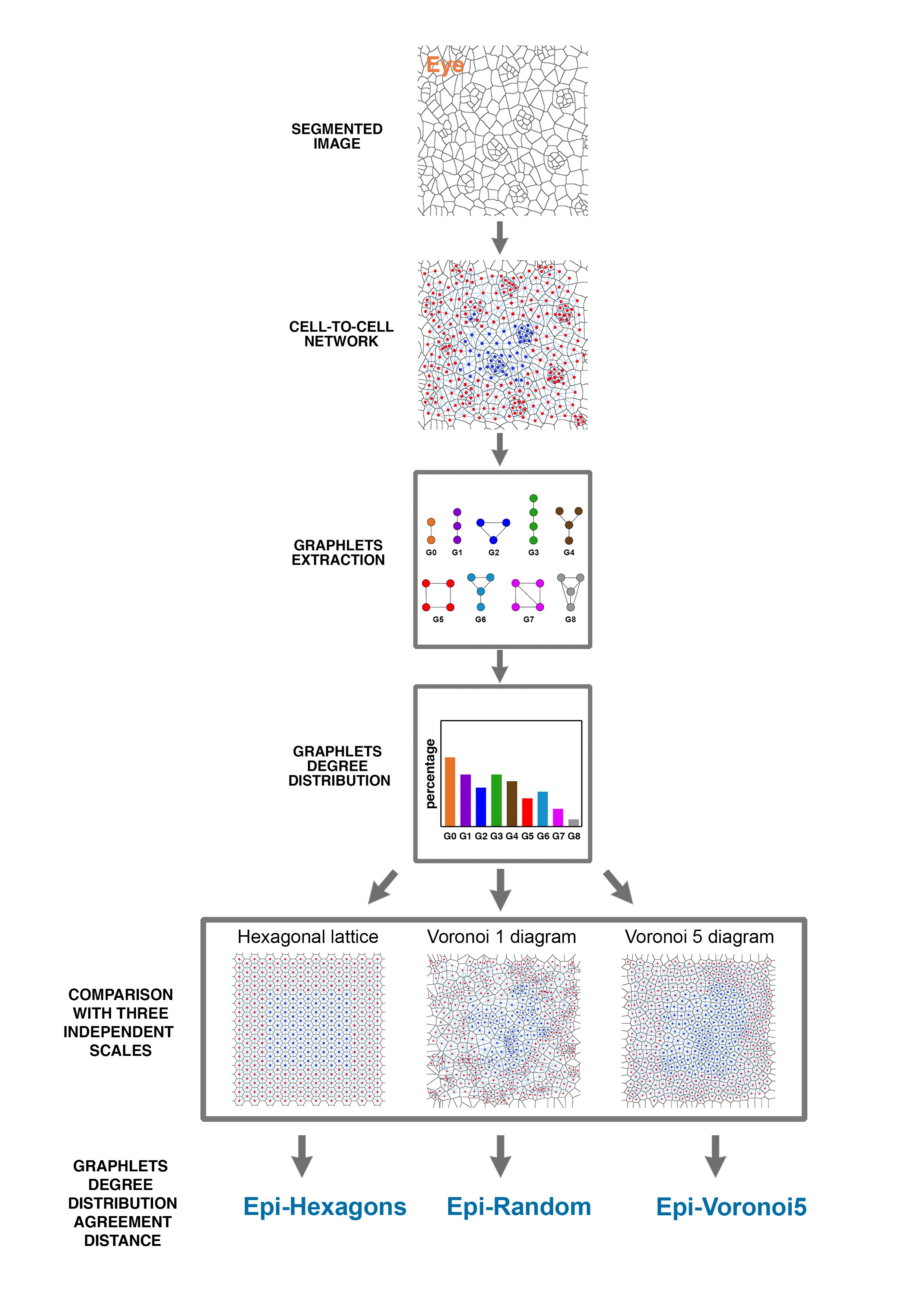

Supplement: btz683_Supplementary_Data [file btz683_supplementary_data.zip › Supplementary Figure 1.jpg]

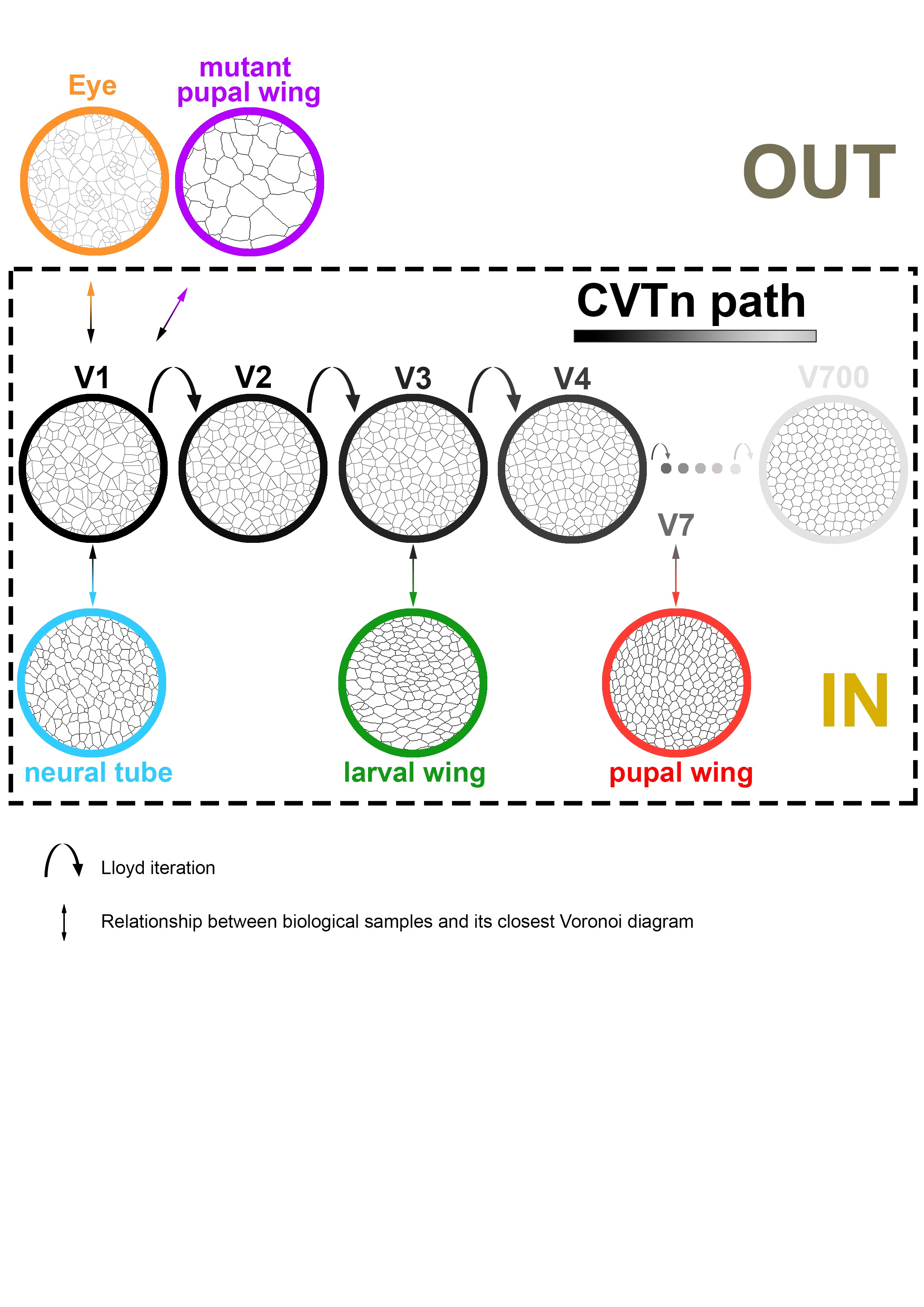

Supplement: btz683_Supplementary_Data [file btz683_supplementary_data.zip › Supplementary Figure 2.jpg]

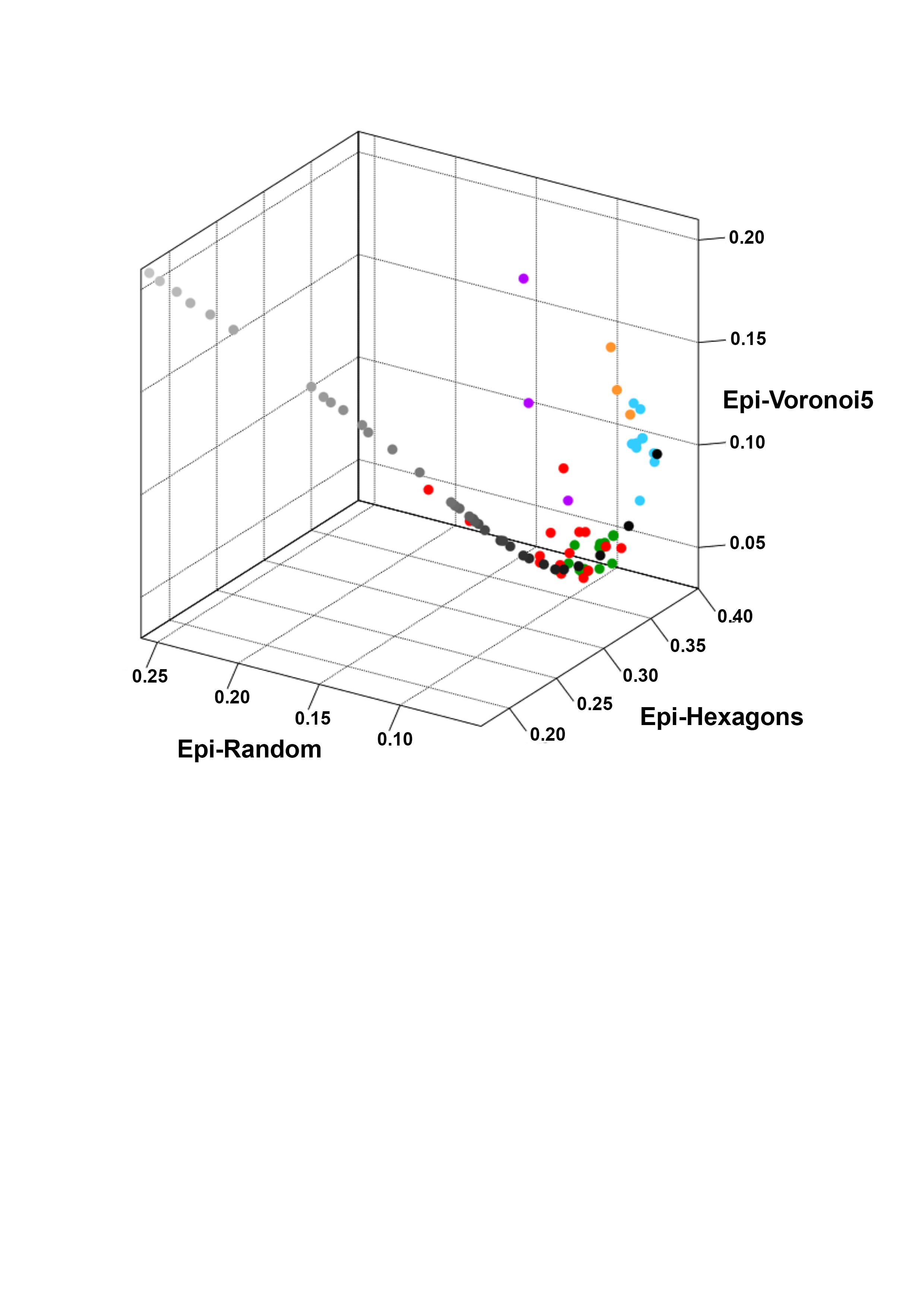

Supplement: btz683_Supplementary_Data [file btz683_supplementary_data.zip › Supplementary Figure 3.jpg]
